# Effect of Plasticizer Type on Tensile Property and In Vitro Indomethacin Release of Thin Films Based on Low-Methoxyl Pectin

**DOI:** 10.3390/polym9070289

**Published:** 2017-07-20

**Authors:** Pensak Jantrawut, Tanpong Chaiwarit, Kittisak Jantanasakulwong, Claire Hélène Brachais, Odile Chambin

**Affiliations:** 1 Department of Pharmaceutical Sciences, Faculty of Pharmacy, Chiang Mai University, Chiang Mai 50200, Thailand; tannahupp@gmail.com; 2 School of Agro-Industry, Faculty of Agro-Industry, Chiang Mai University, Chiang Mai 50100, Thailand; jantanasakulwong.k@gmail.com; 3 ICMUB UMR CNRS 6302, University of Bourgogne Franche-Comté, 9 Avenue Alain Savary, Dijon 21000, France; claire-helene.brachais@u-bourgogne.fr; 4 Department of Pharmaceutical Technology, UMR PAM, Agrosup-University of Bourgogne Franche-Comté, 7 bd Jeanne d’Arc, Dijon 21079, France; odile.chambin@u-bourgogne.fr

**Keywords:** low-methoxyl pectin, indomethacin, glycerin, propylene glycol

## Abstract

This study developed the interests of low-methoxyl pectin (LMP) together with plasticizers for the preparation of elastic thin films. The effect of different plasticizer types (glycerol: Gly; sorbitol: Sor; propylene glycol: PG; and polyethylene glycol 300: PEG 300) and concentrations (20–40% *w/w*) on mechanical and thermal properties of LMP films as well as on in vitro release of indomethacin were evaluated. Without any plasticizer, a brittle LMP film with low tensile strength and % elongation at break was obtained. Addition of plasticizers from 20% to 40% caused reduction in the tensile strength and Young’s modulus values, whereas percent elongation was increased. Forty percent Gly-plasticized and PG-plasticized films were selected to deliver indomethacin in comparison with non-plasticized film. No significant difference in indomethacin release profiles was displayed between the films. The analysis of indomethacin release model indicated that more than one drug release mechanism from the film formulation was involved and possibly the combination of both diffusion and erosion. Even though indomethacin incorporated in non-plasticized film showed similar release profile, Gly or PG should be added to enhanced film flexibility and decrease film brittleness.

## 1. Introduction

Nowadays, concern about limited natural resources and environmental degradation is increasing. A variety of renewable biopolymers has been investigated for development of biodegradable materials to substitute or complement their non-biodegradable petrochemical-based counterparts [1]. Bio-based films are made from natural polymers, of animal or vegetable origin, such as polysaccharides, lipids and proteins. When these materials are released into the environment, they are converted into simple compounds that do not harm the bio-system [2]. Numerous studies have been carried out to investigate the properties of biofilms, made from single hydrocolloid components such as polysaccharides or proteins. The most frequently used polysaccharides were celluloses and starch (and their derivatives), chitosan, seaweed extracts (carrageenans and alginates), exudate (arabic gum), seed (guar gum) or microbial fermentation gums (xanthan and gellan gum), and pectin [3].

Pectin is a polysaccharide that can be extracted from plant cell walls. This natural polymer has low cost, high stability, good gelling property, biocompatibility, non-toxicity and easy modification chemistry and biochemistry. Pectin is composed of linear chains of (1→4)-linked α-d-galacturonic acid residues. The composition of pectin depends on the plant source and conditions employed during pectin isolation and purification. The degree of esterification (DE) and degree of amidation (DA), which are both presented as percentages with respect to the total carboxyl group content, are important in pectin classification. Pectins, in which the galacturonic acid residue is higher than 50%, are known as high-methoxyl pectin (HMP) and those in which the galacturonic acid residue is less than 50% are regarded as low-methoxyl pectin (LMP) [4,5].

In a previous study, films made from pectin were shown as having complete dissolution in water; however, they had poor mechanical properties [6]. Development of pectin films requires just the basic constituents, for instance, pectin as a polymer, solvent and cross-linking agent. Moreover, plasticizers are used in the preparation of the film in order to promote the desired mechanical characteristics. The commonly used plasticizers in carbohydrate-films are polyols such as glycerol and sorbitol. In 2015, Cabello et al. [7] reported the microstructure change of two plasticizers (glycerol and polyethylene glycol) on pectin film. However, the information on drug loaded in pectin films is limited at present.

Indomethacin, which was selected as a model drug, is a non-steroidal anti-inflammatory drug (NSAID) widely used in the treatment of dermatitis and rheumatic diseases by cutaneous route [8]. Although oral therapy with indomethacin is very effective, its clinical use is limited by its potential adverse effects on the gastrointestinal tract. Several studies that aimed at the development of a topical and buccal delivery system of indomethacin to increase local soft tissue and joint concentration have been conducted [9,10,11]. Furthermore, indomethacin is a less soluble drug which makes it a promising candidate for thin film formulation. In this study, different plasticizers including glycerol (Gly), sorbitol (Sor), propylene glycol (PG) and polyethylene glycol 300 (PEG 300), in five concentrations, were tried in pectin films and the effect on tensile and thermal properties and then on indomethacin in vitro release of LMP films were evaluated. 

## 2. Materials and Methods

### 2.1. Materials

Non-amidated low-methoxyl pectin (LMP) (Unipectine OF300C; DE = 30% and DA = 0%) were purchased from Cargill^TM^ (Saint-Germain-en-Laye, France). Glycerol, sorbitol, propylene glycol and polyethylene glycol 300 were used as film plasticizers and were obtained from Srichand Professional (Bangkok, Thailand). Indomethacin was purchased from Sigma Chemical Co. (St. Louis, MO, USA). Calcium chloride (CaCl_2_) and potassium phosphate monobasic (KH_2_PO_4_) were purchased from Merck (Damstadt, Germany). Hydrochloric acid (HCl) 1 M and sodium hydroxide (NaOH) 1 M were purchased from Ajax Finechem (Auckland, New Zealand). Distilled water served as the solvent for preparing the film solutions. All the reagents were analytical grade.

### 2.2. Film Preparation

The LMP-based films were prepared by modified ionotropic gelation with solution-casting techniques. Four plasticizers, which differed in molecular weight and number of hydroxyl (–OH) group, including Gly, Sor, PG and PEG300 were chosen. The film preparation procedures are described as follows: Initially, 3% (*w/w*) aqueous dispersion of LMP was prepared under constant stirring for 30 min at room temperature (25 ± 2 °C). Thereafter, the different plasticizers were added into the dispersions at 20%, 25%, 30%, 35% or 40% (*w/w*, pectin basis). The film-forming solution (4.85 mL) was cast on a 6 cm × 9.7 cm dialysis membrane (Cellu-Sep T3/Nominal MWCO: 12,000–14,000 Da, Membrane Filtration Product, Inc., Seguin, TX, USA). Then the cast film-forming solution on the membranes was placed on crosslinking agent (3% *w/v* CaCl_2_) which was supported by plastic boxes. The gelled film was formed immediately due to the diffusion of calcium cations through dialysis membrane and it was allowed to contact with the cross-linking solution for 3 min. After that, the fresh film was placed on another plastic box and dried in an oven (30 ± 2 °C). All the films were prepared in triplicate including films without any plasticizers which were used as control. After 24 h of drying, film was peeled from the casting surface and stored in a desiccator for further experiments. The film formulations that showed the best tensile properties were selected to load indomethacin in the same total mass of indomethacin in every plasticized films and investigated for in vitro release from the developed film formulation.

### 2.3. Tensile Properties Testing

The solid films were cut into a square shape of 2 cm × 7 cm. The thickness of the films was measured at three different points with a micrometer (GT-313-A, Gotech Testing Machines Inc., Taichung, Taiwan) and the average film thickness was used for each film calculation. Films with nicked sides, cracks, or air bubbles were discarded. The tensile properties of the films were tested using a tensile tester universal tensile machine (Hounsfield Test Equipment, H1KS, Redhill, UK). Film sample was clamped between two tensile grips for film testing and the initial gauge length was set at 5 cm. The film was pulled using a crosshead speed of 2 mm/min. During the stretching, force (N) and elongation at break (mm) were recorded. Only those films that broke at the center of the strip were used for the analysis, and films that broke near the grips were discarded. Six replicate measurements (not including the discarded one) were conducted for each film. The tensile properties were calculated as average value from the obtained results. The tensile properties of the films were characterized by the tensile strength, percent elongation at break and Young’s modulus. These tensile properties were calculated using the following equations:

(1) $$Tensile\;strength\;=\;\frac{F_{max}}{t\;\times w}$$

where *F_max_* is the load at failure (force at which the films break), *t* is the initial film thickness, and *w* is the initial film width.

(2) $$Percent\;elongation\;at\;break\;=\frac{l_{f}\;-l_{0}}{l_{0}}\;\times 100$$

where *l_f_* is the final length of the film at failure and *l*_0_ is the initial length (5 cm) of the film between grips.

(3) Young′s modulus =StressStrain =FAΔll0

where *F* is equal to the force applied to the structure, *A* is the cross-sectional area of the film, Δ*l* is the change in length of the film when the force is applied to it and *l*_0_ is the initial length.

### 2.4. Film Characterization

#### 2.4.1. Morphological Studies

Morphological examination of pectin plasticized thin films and indomethacin incorporated in the selected films was conducted by scanning electron microscopy (SEM) using a JEOL scanning electron microscope (JSM-5410LV, JEOL USA, Inc., Peabody, MA, USA) at 15 kV under low vacuum mode. The films were performed without any coating solution at magnifications ×350. Thickness of films were evaluated.

#### 2.4.2. Determination of Drug Loading

The amount of indomethacin-loaded into the selected film was determined by adding a film which contained 1.78 mg/cm^2^ (6.78 mg) of indomethacin into 100 mL of phosphate buffer (PB; KH_2_PO_4_/NaOH 1 M), pH 7.4 for 4 h until disintegration. The suspension was filtered and the absorbance measured at 320 nm by UV spectroscopy (V530, JUSCO, Tokyo, Japan). Indomethacin content was determined from the standard curve of indomethacin in PB, which was linear with a high correlation coefficient (*r*^2^ = 0.9987). The following regression equation was obtained: *y* = 0.0234*x* + 0.0004, where *y* is the absorbance and *x* is the concentration of indomethacin (mg/L). The experiment was done in triplicate. The percentages of drug loading were calculated according to the following equation:

*Drug loading* (%) = *AQ*/*TQ* × 100 (4)

where *AQ* is the actual quantity of indomethacin present in the film and *TQ* is the theoretical quantity of indomethacin.

#### 2.4.3. Water Content Determination

The water content of pectin-plasticized thin films and indomethacin incorporated in the selected films was determined using a moisture analyzer at 100 °C until stabilization of weight was achieved (OHAUS MB35, Greifensee, Switzerland).

### 2.5. Differential Scanning Calorimetry (DSC)

The films were analyzed using differential scanning calorimetry (DSC Q1000, TA Instrument, New Castle, DE, USA). The calibration of the equipment was conducted using indium as standard (*T*_m_ = 156.6 °C). The samples were conditioned at 55% RH, 25 °C for 48 h before DSC measurement. For differential scanning calorimetry (DSC) analyses, 10 mg of film samples were weighted and placed in an aluminum sample pan (TA Instrument, New Castle, DE, USA) which was immediately sealed. An empty sample pan was used as reference. Film samples were heated from −90 to 250 °C at a rate of 10 °C/min. Nitrogen gas was used to flush the DSC cell at a flow rate of 50 mL/min to maintain an inert environment. Experiments were done in triplicate.

### 2.6. Fourier-Transform Infrared Spectroscopy (FT-IR)

FT-IR experiments were conducted using a Thermo Scientific Nicolet IS10 spectrometer (Thermo Fisher Scientific, Waltham, MA, USA) by a reflection technique (ATR). The absorbance data were processed for the wave number range of 400–4000 cm^−1^ using the software OMNIC Spectra 8.3.103 (Thermo Fisher Scientific, Waltham, MA, USA). Each film sample was respectively deposited directly between the two crystals. FT-IR absorbance spectra were also obtained for pectin and indomethacin powders for comparison with plasticized and indomethacin-loaded films.

### 2.7. In Vitro Indomethacin Release

The in vitro indomethacin release experiments were conducted using a small paddle apparatus (200 mL cylindrical vessel) equipped with enhancer-cell (Enhancer cell, Varian Inc., North Carolina 27513-2250, Palo Alto, CA, USA). An enhancer cell comprises a 2.01 cm^2^ surface area. Each film sample was cut into pieces with diameter 2 cm (with mean indomethacin amount of 1.18 mg/cm^2^) and fixed by the retaining ring to the cell body. The top side of the enhancer cell was placed in the dissolution chamber which was filled with 100 mL Tris buffer pH 7.4 (Tris/HCl 1 M). The dissolution apparatus was operated with a paddle stirrer at 50 rpm rotation speed and a temperature of 32.0 ± 0.2 °C was maintained throughout the experiment, mimicking the skin temperature. The samples were withdrawn at different time intervals up to 24 h and replenished with an equal volume of Tris buffer solutions at each time interval. The absorbance of the withdrawn samples was measured at 320 nm using UV spectrophotometer (Cary 50Bio UV–Visible Spectrophotometer, Orsay, France). All the dissolution runs were performed in triplicate. Non-plasticized film without indomethacin was used as control to assess that no other compound absorbs at 320 nm.

To analyze the drug release rate kinetics and mechanism of drug release, the in vitro dissolution data were fitted into Zero-order, Higuchi matrix model and Korsmeyer–Peppas empirical power law in order to obtain the best-fit model.

(a) Zero-order kinetics: The release rate data were fitted into the following equation,
  (5) $$Q_{t}=\;Q_{o}+K_{o}\times \;t$$
  where *Q_t_* is the amount of drug dissolved in time (*t*), *Q_o_* is the initial amount of drug in the solution, and *K_o_* is the zero-order release constant.
(b) Higuchi matrix model: The release rate data were fitted to the following equation,
  (6) $$Q_{t}=\;K_{H}\times \;t^{1/2}$$
  where *Q_t_* is the amount of drug released in time (*t*), *K_H_* is the Higuchi diffusion constant.
(c) Korsmeyer–Peppas empirical power law:
  (7) $$M_{t}/M_{8}=\;Kt_{n}$$
  where, *M_t_*/*M*_8_ is the fraction of drug released at a time (*t*), *K* is the structural and geometrical constant and *n* is the release exponent.

### 2.8. Statistical Analysis

All the data were presented as mean ± SD. One way ANOVA was used to evaluate the significance of differences at the significant level of *p*-value < 0.05. Statistical analysis was performed using SPSS software version 16.0 (SPSS Inc., Chicago, IL, USA).

## 3. Results and Discussion

### 3.1. Film Preparation

When low-methoxyl pectin (LMP) film forming solution was placed onto a solution containing CaCl_2_, calcium ions were permeated through the Cellu-Sep membrane. Then, the gelled film was formed by ionotropic gelation mechanism in which intramolecular cross-links occurred between negatively charged carboxyl groups of LMP and the positively charged counter ion (Ca^2+^). The films prepared by 3% *w/w* LMP cross-linked with 3% CaCl_2_ were homogeneous, transparent with slightly hazy appearance and easily removed from the plastic cast plates after 10 min of cooling at room temperature (suitable film handling). However, the films containing less than 3% *w/w* of LMP and 2% *w/v* cross-linking agent (CaCl_2_) were very brittle and were not tested in this study. More flexible films were obtained when a plasticizer was added. Moreover, LMP films prepared using the cross-linking method were colorless compared to those obtained with high-methoxyl pectin (slight yellowness) prepared by casting method [12].

### 3.2. LMP Films Characterization 

**Figure 1:**
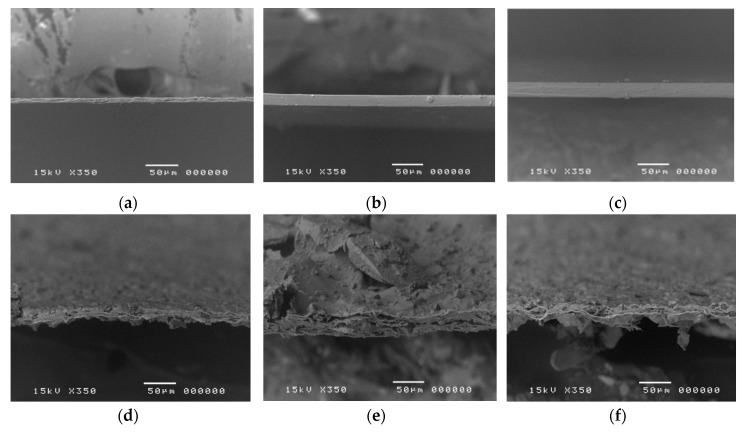
Scanning electron microscopy of the thickness at 350 × magnifications of non-plasticized (**a**) 40% Gly-plasticized (**b**) and 40% PG-plasticized (**c**) LMP films and those films loaded indomethacin (**d**–**f**), respectively. Gly: Glycerol; PG: Propylene glycol; LMP: Low-methoxyl pectin.

LMP films with and without plasticizer exhibited a colorless, translucent and smooth surface. Scanning electron microscopy images of non-plasticized, 40% Gly-plasticized and 40% PG-plasticized LMP films were shown in Figure 1. The thicknesses of non-plasticized, 40% Gly-plasticized and 40% PG-plasticized LMP films without indomethacin were around 10, 25 and 28 µm, respectively (Figure 1a–c). The thickness was observed to increase when plasticizer was added. When indomethacin was loaded, opaque white films and rougher surfaces of indomethacin particles were observed (Figure 1d–f). Generally, the morphology of the film should appear homogeneous and continuous to ensure uniform distribution of drug throughout the polymeric mixture. However, self-aggregation might occur during drying because of the intermolecular and convective forces, leading to wrinkled surface in films especially when drug was added. Additionally, interaction between drug and polymers, and the crystalline nature of the drug, may cause the formation of a rough surface in films [13]. The morphological state of the film may impact the tensile properties, for example, by crystal growth. Therefore, these aspects have to be considered during the development of films for pharmaceutical use [14].

All the LMP film formulations exhibited fairly uniform drug content with 100% of indomethacin content (Table 1). No significant difference of drug contents was observed between non-plasticized and plasticized (Gly and PG) LMP films. In the case of poor aqueous solubility compounds (like indomethacin), high drug content may be obtained using the dialysis membrane production method. Preparation of indomethacin-loaded film by membrane method involves the diffusion of ions (Ca^2+^) across the membrane, causing pectin film formation and leaving a homogeneous dispersion of the drug in the gel. The percentage of water content in LMP film-loaded indomethacin was shown in Table 1. 40% PG-plasticized and Gly-plasticized LMP films showed higher water content (9.60% and 9.54%, respectively) than non-plasticized LMP films-encapsulated indomethacin (9.05%). This response may be attributed to the hygroscopic nature of glycerol and propylene glycol in the films. The hydrophilic and hygroscopic nature of plasticizers actually forms the large hydrodynamic plasticizer–water complex [15]. However, the equilibrium moisture content of films was not found to be significantly affected by the plasticizer type. 

### 3.3. Mechanical Properties

#### 3.3.1. Tensile Strength at Break of Pectin Plasticized Films

**Figure 2:**
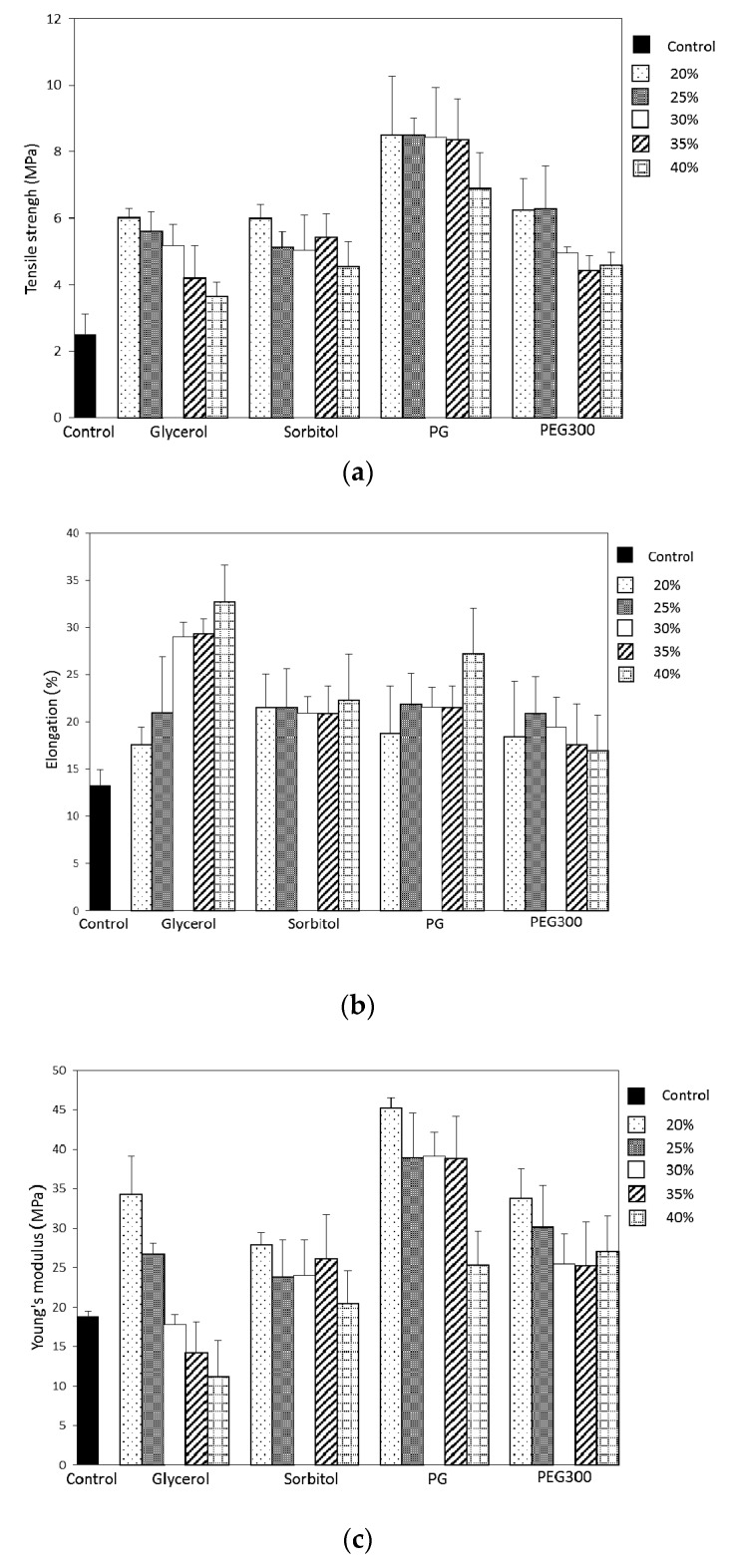
Effect of plasticizer type and concentration on the tensile strength (**a**), elongation (%) at break (**b**) and Young’s modulus (**c**) of LMP films.

The effect of different plasticizer types and concentrations on the tensile strength at break of pectin films was shown in Figure 2a. The presence of plasticizers at a low concentration of 20% demonstrated high tensile strength value of 6.01, 5.11, 8.51 and 6.24 MPa for Gly-plasticized, Sor-plasticized, PG-plasticized and PEG-plasticized films, respectively. Without any plasticizer (blank), a brittle film with a low tensile strength value of 2.49 MPa was obtained. The possible reason for the high tensile strength for low plasticizer concentration is the domination of strong hydrogen bonds produced by pectin–pectin intermolecular interactions over pectin–plasticizer attraction. However, addition of plasticizers at concentrations from 20% to 40% caused significant reduction in the tensile strength of Gly-plasticized and PG-plasticized films. As the plasticizer concentration increased from 20% to 40%, Gly-plasticized films exhibited the highest reduction value of 39% (decreased to 3.66 MPa) in tensile strength followed by PG-plasticized films with reduction value of 19% (dropped to 6.90 MPa). As far as Sor-plasticized and PEG-plasticized films are concerned, there was observed reduction in tensile strength, but it was not significantly different. Decrease in the tensile strength of pectin-based films with increase in the concentration of the plasticizer has been reported by many authors [12,16,17]. In general, plasticizer molecules with smaller molar mass can facilitate easy interaction between plasticizer–polymer molecular chains [18], but these results revealed that glycerol (92.09 g/mol) has higher efficiency in plasticizing pectin films as compared to propylene glycol (76.09 g/mol), sorbitol (182.17 g/mol), and polyethylene glycol 300 (285–315 g/mol). Other researchers have also reported a similar observation with other polysaccharides where glycerol induced greater tensile strength reduction compared to other polyols [19,20].

#### 3.3.2. Elongation at Break of Pectin Plasticized Films

The effect of plasticizer concentration (20–40%) on the elongation at break of pectin plasticized films was shown in Figure 2b. Elongation of pectin film without any plasticizer (blank) was 13.24%. The increasing of plasticizer concentration from 20% to 40% led to a significant increase in the film elongation: 17.56% to 32.75% for Gly-plasticized films. For Sor-plasticized and PG-plasticized films, the elongation increased from 21.52% to 22.31% and from 18.80% to 27.23%, respectively. However, the elongation of PEG-plasticized films decreased from 18.47% to 16.95%, when plasticizer content increased from 20% to 40%. Elongation at break is defined as the ability of film to deform before finally breaking. This parameter (% elongation) helps to determine the flexibility and stretch ability of films. Elongation of polymeric materials depends on the mobility of their molecular chains. The increasing in films elongation can be explained by the fact that plasticizers decrease the intermolecular bonds between polymer matrixes and substitute them with hydrogen bonds formed between plasticizer and polymer molecules. Such disruption and reconstruction of polymer molecular chains reduce the rigidity and promote the flexibility of films by allowing more chain mobility [20,21]. Generally, a soft and weak polymer such as pectin is identified with low tensile strength and low elongation at break values [22]. Humectant-plasticizers such as glycerol and propylene glycol were used because they act together with water to promote softness and flexibility. However, high concentration of 40% PEG300 might lead to phase separation, which, as a consequence, exhibited lower elongation at break.

#### 3.3.3. Young’s Modulus of Pectin Plasticized Films

The Young’s modulus stands for the resistance of the film to elastic deformation and this can be perceived as reflecting the stiffness and strength of the film [23,24]. Low Young’s modulus value corresponds to flexible film. The effect of plasticizer concentration (20–40%) on the Young’s modulus values of pectin plasticized films was shown in Figure 2c. Young’s modulus values regularly decreased with the addition of plasticizer content, meaning that LMP films lost their stiffness and became more flexible with the addition of the plasticizer. Among the plasticizers tested, glycerol was the most effective plasticizer in depressing the Young’s modulus values (down to 11.19 MPa). At 40% *w/w* plasticizer, LMP films containing PEG300 exhibited the highest Young’s modulus value of 27.09 MPa. Moreover, a small variation of Young’s modulus values was observed with PEG300 whatever the amount of incorporated plasticizer. These results, together with mechanical properties at break presented before, tend to confirm that PEG300 is not able to establish quantitative specific interactions with pectin matrix. Thus, this polyether oligomer cannot be considered as an efficient plasticizer for pectin film. LMP film without plasticizer (blank) also presented low Young’s modulus value (18.81 MPa). Although the pectin films did not show tensile properties like a large number of other polymeric films such as starch and gelatin films, the plasticized−pectin films were observed to improve their stiffness, tensile strength and flexibility. These results revealed that 40% Gly-plasticized LMP film with the best elongation property enhanced the LMP film flexibility, decreased the brittleness and would be able to avoid tearing during handling and storage.

From this study, glycerol and propylene glycol appear to be the best plasticizer for pectin film. From this reason, two indomethacin-loaded film formulations composed of 40% glycerol (Gly-) and propylene glycol (PG-) as plasticizer were prepared. Then, the indomethacin-loaded films were investigated for mechanical properties, thermal analysis as well as in vitro release.

#### 3.3.4. Mechanical Properties of Pectin-Loaded Films

The mechanical properties of film formulations containing a drug are a crucial factor not only during the production or development, but also regarding the proper handling by the patient. Thus, the effect of the incorporation of the drug in the plasticized matrix can be interesting. Tensile strength, % elongation at break and Young’s modulus values of LMP films containing indomethacin were presented in Table 1. For example, % elongation at break of non-plasticized, 40% Gly-plasticized and 40% PG-plasticized films that loaded indomethacin were 10.91%, 26.50% and 21.17%, compared to 13.24%, 32.75% and 27.23% for films without indomethacin, respectively. The plasticizer–polymer interactions may be interrupted by the incorporated indomethacin and reduced the film elongation. Indomethacin has functional groups such as benzoyl (C=O) and carboxylic (–COOH) groups. These groups may also interact with pectin chain resulting in the increasing of Young’s modulus value.

### 3.4. DSC Analysis

**Figure 3:**
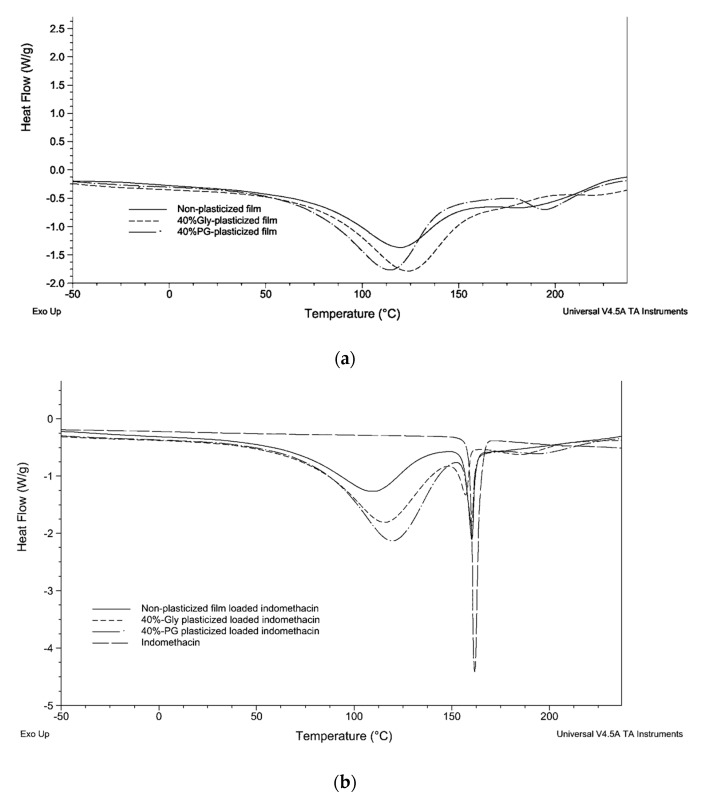
Differential scanning calorimetry (DSC) curves of pectin films with and without plasticizer (**a**) and with indomethacin (**b**).

DSC curves of plasticized and non-plasticized LMP pectin films are presented in Figure 3. When pectin films were analyzed between −50 and 175 °C, no *T*_g_ nor melting peak were observed, which indicated the totally amorphous structure of the films. However, a broad endothermic peak was observed around 115 °C. This endothermic behavior is linked to the evaporation of water entrapped in the pectin matrices [25,26]. The changes in enthalpy (Δ*H*) in endothermic peaks which are calculated by integrating the areas below the DSC curves and endothermic peak temperatures (EPTs) are shown in Table 2. EPTs of non-plasticized, Gly-plasticized and PG-plasticized films without indomethacin were not significantly different. For 40% PG-plasticized film, the peak around 190 °C, which was propylene glycol boiling temperature, was observed (Figure 3a). Pectin films, when incorporating a plasticizer (Gly or PG), were found to absorb more thermal energy (371.2 ± 7.6 and 390.4 ± 6.2 J/g, respectively) than pectin films without any plasticizers (238.8 ± 1.8 J/g). The increase in Δ*H* values of plasticized films may be due to the hygroscopic character of both glycerol and propylene glycol, which tends to provide additional water into the film. The plasticizer type affects significantly the changes in enthalpy since propylene glycol seems to promote water incorporation in pectin films. If the amount of water in the film is proportionally correlated to the number of hydrogen bonds between the pectin matrix, the plasticizer and water molecules, then, the high amount of water incorporated in PG-plasticized films may explain the highest Young’s modulus values of PG-plasticized films.

It was found that when indomethacin was added into the films, Δ*H*s values of endothermic water peaks in pectin matrices systematically decreased compared to the Δ*H*s values for non-loaded films (Figure 3b). This might be due to the hydrophobic character of indomethacin preventing the absorption of water molecules in the films. Whereas EPTs of non-loaded films were not significantly different, EPTs of water evaporation for loaded films range between 109.5 and 119.2 °C. DSC curves of indomethacin-loaded pectin films have also been exhibited as a sharp peak at about 159 °C [27] which demonstrated the presence of crystallized indomethacin in the films. There was no significant difference between EPT of indomethacin crystalline melting peak of non-plasticized, 40% Gly-plasticized and 40% PG-plasticized films loaded indomethacin. This crystalline amount of drug supported the white aspect of films containing indomethacin. Interestingly, the Δ*H* of indomethacin crystalline melting peak was significantly decreased when Gly (21.3 ± 4.5 J/g) or PG (25.7 ± 2.2 J/g) was added in the films compared with indomethacin film without any plasticizers (39.9 ± 1.6 J/g). This Δ*H* result revealed that addition of 40% *w/w* glycerol or propylene glycol reduced the crystallinity of indomethacin and certainly promoted the amount of amorphous drug in the plasticized films.

### 3.5. FT-IR Spectroscopic Study

The FT-IR spectra of pectin powder, pectin films and indomethacin were recorded in order to eventually identify specific interactions between the drug and/or the matrix and the plasticizers. Pectin powder showed ester carbonyl bands at 1731 cm^−1^ and the characteristic ether C–O band at 1014 cm^−1^ as well as carboxylate (–COO^−^) bonds at 1605 cm^−1^. The bond at ~1599–1602 cm^−1^ was much stronger when pectin film was prepared. The gel formation in film-forming process involves the simultaneous bonding of calcium ions to carbonyl groups of the LMP pectin molecules. An egg-box-like model was proposed to explain the structure of pectin molecules bound by the calcium ions [28]. Therefore, carboxylate groups in the pectin films are significantly responsible for egg-box-like model which were formed by ionotropic gelation. Several characteristic absorption bands are detected on the FT-IR spectrum of indomethacin and some specific bonds which can be used as marker peak of indomethacin are indicated as follow: 1712 cm^−1^ (C=O of carboxylic acid), 1689 cm^−1^ (benzoyl C=O), 1306 cm^−1^ (C–O of acidic group) and 1067 cm^−1^ (C–Cl) [29,30]. All these marker peaks especially the one accounting for the C–Cl bond (~1068 cm^−1^) appeared for all indomethacin-loaded pectin films. Moreover, no shift of the acidic carbonyl band (1712 cm^−1^) could be detected when indomethacin was incorporated in the plasticized films. This observation means that no specific appeared between the drug and the plasticizers in these films.

### 3.6. In Vitro Release of Indomethacin 

**Figure 4:**
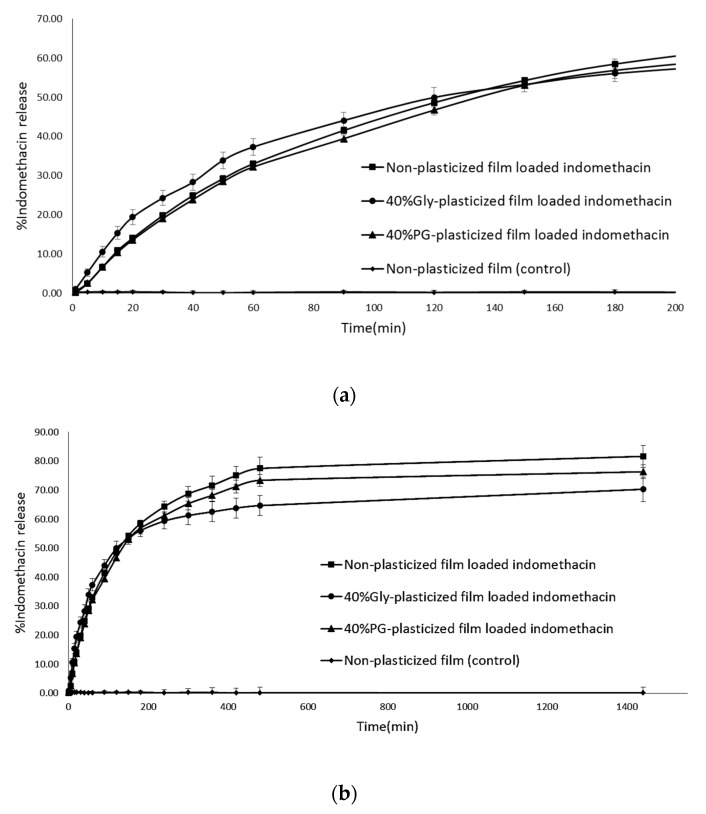
Dissolution profiles of LMP films-loaded indomethacin in Tris buffer pH 7.4 at different time intervals from 0 to 200 min (**a**) and up to 24 h (**b**).

The enhancer cell which is made of Teflon^®^, an inert and non-reactive material, was selected to study the drug release profiles to present always the same film surface to the release medium. Indomethacin release from three film formulations including non-plasticized, Gly-plasticized and PG-plasticized was assessed in vitro in Tris buffer. The dissolution profiles from the films in the dissolution media are presented in Figure 4. The same dissolution profile pattern was observed when indomethacin was loaded in different film formulations with two steps; initially, the phase corresponding to the release of indomethacin on the film surface, followed by a second phase corresponding to slow release of the drug from the matrix. More than 50% of indomethacin release in all formulations was achieved after 120 min. The order of indomethacin released from LMP film formulations was as follows: Non-plasticized LMP film > 40% PG-plasticized film > 40% Gly-plasticized film in Tris buffer. However, there was no significant difference (*p* > 0.05) between the formulations. To analyze the mechanism of indomethacin release from LMP films, the drug release data were fitted to various kinetics models as for in vitro dissolution for drug delivery [31]. When the cumulative amount of drug released was plotted against time, the permeation profiles of the drug were found to follow zero-order kinetics. Further, in order to find out whether diffusion or erosion was involved in the drug release. The data were subjected to analysis using the Higuchi and Korsmeyer–Peppas models, which plotted cumulative percentage of drug released versus square root or log of time, respectively. The model that fits the release data was evaluated by correlation coefficient (*r*). The *r* values were used as the criteria to choose the best model to describe drug release from the controlled release systems. The *r*^2^ values of all samples were higher in the Higuchi model (0.9985, 0.9910 and 0.9977) than in the zero-order model (0.9491, 0.9098 and 0.9477) and the Korsmeyer–Peppas model (0.9659, 0.9732 and 0.9694) (Table 3). This result indicated that indomethacin release from LMP films followed the diffusion-controlled matrix model. The *n* values obtained using the Korsmeyer–Peppas equation were between 0.6921 and 0.8881 for all formulations. These values are characteristic of anomalous kinetics (non-Fickian), suggesting that more than one drug release mechanism may be involved in release kinetics besides, possibly a combination of both diffusion and erosion [32]. At the end of the experiment, the state of the film was noted and they were not damaged but sometimes they presented small blisters due to water uptake and may be at the beginning of erosion.

## 4. Conclusions

The choice and design of polymers and plasticizers in drug delivery systems are crucial for drug release characteristics as well as for mechanical and thermal properties of the formulation. In this study, tensile properties of LMP film with the addition of four different plasticizers at different concentrations were tested. Plasticized films presented improved handling properties when compared to the films designed without any plasticizers. When increasing the plasticizer amounts, all four plasticizers tended to decrease the tensile strength at break and Young’s modulus and increased the percent elongation of the films. Among the plasticizers tested, glycerol is the most efficient plasticizer in changing the tensile properties and the appearance of the film, preventing film cracking and increasing film flexibility. LMP Gly-plasticized and PG-plasticized films containing indomethacin as a model drug were prepared and the influence of plasticizers on tensile, thermal properties and indomethacin release was investigated. There was no significant difference in in vitro indomethacin release between non-plasticized, Gly-plasticized and PG-plasticized LMP films. However, plasticizers such as glycerol and propylene glycol should be added in order to get LMP films that are more flexible and less susceptible to brittleness. The developed films are also requiring future studies to evaluate their skin toxicity and/or irritation.

## Figures and Tables

**Table 1:** Mechanical properties, drug content and water content of non-plasticized, 40% Gly-plasticized and 40% PG-plasticized LMP films containing indomethacin.

Indomethacin Loaded LMP Films	Tensile Strength (MPa)	Elongation (%)	Young’s Modulus (MPa)	Drug Loading (%)	Water Content (%)
Non-plasticized	2.57 ± 0.62	10.91 ± 1.14	21.80 ± 2.38	103.59 ± 6.56	9.05 ± 0.36
40% Gly-plasticized	3.26 ± 0.08	26.50 ± 3.86	23.41 ± 4.71	100.96 ± 10.10	9.54 ± 1.83
40% PG-plasticized	4.41 ± 0.45	21.17 ± 2.27	32.44 ± 5.35	100.83 ± 9.80	9.60 ± 1.86

**Table 2:** Endothermic peak temperatures (EPT) and change in enthalpy in the endothermic process (Δ*H*) of non-plasticized, 40% Gly-plasticized and 40% PG-plasticized LMP films without and with indomethacin.

Sample	Water Evaporation Peak		Indomethacin Crystalline Melting Peak	
	Δ*H* (J/g)	EPT (°C)	Δ*H* (J/g)	EPT (°C)
Non-plasticized film	238.8 ± 1.8	116.8 ± 2.7	-	-
40% Gly-plasticized film	371.2 ± 7.6	120.3 ± 3.9	-	-
40% PG-plasticized film	390.4 ± 6.2	111.9 ± 6.6	-	-
Non-plasticized film containing indomethacin	191.6 ± 12.3	109.5 ± 1.1	39.9 ± 1.6	159.9 ± 0.6
40% Gly-plasticized film containing indomethacin	235.3 ± 18.3	114.4 ± 0.3	21.3 ± 4.5	158.0 ± 1.2
40% PG-plasticized film containing indomethacin	310.6 ± 19.9	119.2 ± 0.1	25.7 ± 2.2	160.0 ± 0.3
Indomethacin	-	-	106.4 ± 0.4	161.9 ± 0.3

**Table 3:** Release kinetic data of the LMP film containing indomethacin.

Sample	Zero-Order		Higuchi		Korsmeyer–Peppas	
	*r*^2^	*K*_0_ (*h*^−1^)	*r*^2^	*K*_H_ (*h*^1/2^)	*r*^2^	*n*
Non-plasticized film containing indomethacin	0.9491	4.4360	0.9985	9.8634	0.9659	0.6921
40% Gly-plasticized film containing indomethacin	0.9098	8.3395	0.9910	4.781	0.9732	0.8175
40% PG-plasticized film containing indomethacin	0.9477	4.4795	0.9977	9.2185	0.9694	0.8881

Note: *K*_0_ is the zero-order rate constant expressed in units of concentration/time in hours. *K*_H_ is the rate constant for the Higuchi rate equation. The *n* value, diffusion exponent, is used to characterize different release mechanisms for cylindrical shaped.

## References

[1] Alves V.D., Costa N., Coelhoso I.M. (2010). Barrier properties of biodegradable composite films based on kappa-carrageenan/pectin blends and mica flakes. Carbohydr. Polym..

[2] Chandra R., Rustgi R. (1998). Biodegradable polymers. Prog. Polym. Sci..

[3] Krochta M., Johnston C.D. (1997). Edible and biodegradable polymer films: Challenges and opportunities. Food Technol..

[4] Ridley B.L., O’Neill M.A., Mohnen D.A. (2001). Pectins: Structure, biosyntbesis and oligogalacturonide-related signaling. Phytochemistry.

[5] Rolin C., Whistler R.L., Bemiller J.N. (1993). Pectin. Industril Gums: Polysaccharides and Their Derivatives.

[6] Seixasa F.L., Turbianib F.R.B., Salomãob P.G., Souzaa R.P., Gimenes M.L. (2013). Biofilms composed of alginate and pectin: Effect of concentration of crosslinker and plasticizer agents. Chem. Eng. Trans..

[7] Cabello P.S.D., Takara E.A., Marchese J., Ochoa N.A. (2015). Influence of plasticizers in pectin films: Microstructural changes. Mater. Chem. Phys..

[8] Kwan K.C., Breault G.O., Umbenhauer E.R., McMahon F.G., Duggan D.E. (1976). Kinetics of indomethacin absorption, elimination, and enterohepatic circulation in man. Eur. J. Pharm. Biopharm..

[9] Deore V.A., Kumar R.S., Gide P.S. (2013). Development and statistical optimization of mucoadhesive buccal patches of indomethacin: In Vitro and ex vivo evaluation. Int. J. Adv. Pharm. Biol. Chem..

[10] Puglia C., Trombetta D., Venuti V., Saija A., Bonina F. (2004). Evaluation of in vitro topical anti-inflammatory activity of indomethacin from liposomal vesicles. J. Pharm. Pharmacol..

[11] Miyazaki S., Takahashi A., Kubo W., Bachynsky J., Lobenberg R. (2003). Poly *N*-butylcyanoacrylate (PNBCA) nanocapsules as a carrier for NSAIDs: In vitro release and in vivo skin penetration. J. Pharm. Pharm. Sci..

[12] Pérez C.D., Flores S.K., Marangoni A.G., Gerschenson L.N., Rojas A.M. (2009). Development of a high methoxyl pectin edible film for retention of l-(+)-ascorbic acid. J. Agric. Food. Chem..

[13] Hermans K., Van Den Plas D., Kerimova S., Carleer R., Adriaensens P., Weyenberg W., Ludwig A. (2014). Development and characterization of mucoadhesive chitosan films for ophthalmic delivery of cyclosporine A. Int. J. Pharm..

[14] Preis M., Knop K., Breitkreutz J. (2014). Mechanical strength test for orodispersible and buccal films. Int. J. Pharm..

[15] Krochta J.M., Gennadios A. (2002). Proteins as raw materials for films and coatings: Definitions, current status, and opportunities. Protein-Based Films and Coatings.

[16] Hiorth M., Tho I., Sande S.A. (2003). The formation and permeability of drugs across free pectin and chitosan films prepared by a spraying method. Eur. J. Pharm. Biopharm..

[17] Hoagland P.D., Parris N. (1996). Chitosan/pectin laminated films. J. Agric. Food Chem..

[18] Tapia-Blácido D.R., Sobral P.J.D.A., Menegalli F.C. (2013). Effect of drying conditions and plasticizer type on some physical and mechanical properties of amaranth flour films. LWT Food. Sci. Technol..

[19] Razavi S.M.A., Amini A.M., Zahedi Y. (2015). Characterisation of a new biodegradable edible film based on sage seed gum: Influence of plasticiser type and concentration. Food Hydrocoll..

[20] Muscat D., Adhikari B., Adhikari R., Chaudhary D.S. (2012). Comparative study of film forming behaviour of low and high amylose starches using glycerol and xylitol as plasticizers. J. Food. Eng..

[21] Fishman M.L., Coffin D.R., Konstance R.P., Onwulata C.I. (2000). Extrusion of pectin/starch blends plasticized with glycerol. Carbohydr. Polym..

[22] Bharkatiya M., Nema R.K., Bhatnagar M. (2010). Designing and characterization of drug free patches for transdermal application. Int. J. Pharm. Sci. Drug Res..

[23] Omelczuk M.O., McGinity J.W. (1992). The influence of polymer glass transition temperature and molecular weight on drug release from tablets containing poly(dl)-lactic acid. Pharm. Res..

[24] Roberts R.J., Rowe R.C. (1987). The Young’s modulus of pharmaceutical materials. Int. J. Pharm..

[25] Einhorn-Stoll U., Kunzek H. (2009). The influence of storage conditions heat and humidity on conformation, state transitions and degradation behavior of dried pectins. Food. Hydrocoll..

[26] Iijima M., Nakamura K., Hatakeyama T., Hatakeyama H. (2000). Phase transition of pectin with sorbed water. Carbohydr. Polym..

[27] Budavari S. (1996). The Merck Index—An Encyclopedia of Chemicals, Drugs, and Biological.

[28] Grant G.T., Morris E.R., Rees D.A., Smith P.J.C., Thom D. (1973). Biological interactions: The egg box model. FEBS Lett..

[29] Umeda Y., Fukami T., Furuishi T., Suzuki T., Makimura M., Tomono K. (2007). Molecular complex consisting of two typical external medicines: Intermolecular interaction between indomethacin and lidocaine. Chem. Pharm. Bull..

[30] Del Arco M., Cebadera E., Gutiérrez S., Martín C., Montero M.J., Rives V., Rocha J., Evilla M.A. (2004). Mg, Al layered double hydroxides with intercalated indomethacin: Synthesis, characterization, and pharmacological study. J. Pharm. Sci..

[31] Siepmann J., Siepmann F. (2008). Mathematical modeling of drug delivery. Int. J. Pharm..

[32] Arora G., Malik K., Singh I., Arora S., Rana V. (2011). Formulation and evaluation of controlled release matrix mucoadhesive tablets of domperidone using Salvia plebeian gum. J. Adv. Pharm. Technol. Res..

